# Liquid biopsy in pediatric brain tumors

**DOI:** 10.3389/fgene.2022.1114762

**Published:** 2023-01-06

**Authors:** Arushi Tripathy, Vishal John, Jack Wadden, Seongbae Kong, Sana Sharba, Carl Koschmann

**Affiliations:** ^1^ Department of Neurosurgery, Michigan Medicine, Ann Arbor, MI, United States; ^2^ Department of Pediatrics, Michigan Medicine, Ann Arbor, MI, United States

**Keywords:** liquid biopsy, pediatric, brain tumor, cell-free tumor DNA (cf-tDNA), cerebrospinal fluid (CSF), plasma, targeted treatment

## Abstract

Malignant primary brain tumors are the most common cancer in children aged 0–14 years, and are the most common cause of death among pediatric cancer patients. Compared to other cancers, pediatric brain tumors have been difficult to diagnose and study given the high risk of intracranial biopsy penetrating through vital midline structures, where the majority of pediatric brain tumors originate (Ostrom et al., 2015). Furthermore, the vast majority of these tumors recur. With limitations in the ability to monitor using clinical and radiographic methods alone, minimally invasive methods such as liquid biopsy will be crucial to our understanding and treatment. Liquid biopsy of blood, urine, and cerebrospinal fluid (CSF) can be used to sample cfDNA, ctDNA, RNA, extracellular vesicles, and tumor-associated proteins. In the past year, four seminal papers have made significant advances in the use of liquid biopsy in pediatric brain tumor patients (Liu et al., 2021; Cantor et al., 2022; Miller et al., 2022; Pagès et al., 2022). In this review, we integrate the results of these studies and others to discuss how the newest technologies in liquid biopsy are being developed for molecular diagnosis and treatment response in pediatric brain tumors.

## Introduction

The most common cause of death among pediatric cancer patients aged 0–14 years are malignant primary brain tumors (Ostrom et al., 2020). Unfortunately, diagnosis *via* clinical evaluation and MRI imaging is uncertain, with a differential diagnosis including abscesses and tumor variants requiring discordant treatments. Therefore, current clinical decision-making requires surgical biopsy or resection for histopathologic diagnosis. Even the most minimally invasive option–stereotactic needle biopsy–puts the patient at risk of side effects of general anesthesia, intracranial hemorrhage, neurologic deficit, and death (Nishihara et al., 2011; Hamisch et al., 2017; He et al., 2021). Furthermore, tissue biopsy samples a single point, failing to capture intratumoral heterogeneity for accurate diagnosis (Ng and Lim, 2008). It has previously been shown that neuropathologic diagnosis *via* stereotactic biopsy specimen *versus* tumor resection specimen of the same lesion differed in approximately 40% of cases (Jackson et al., 2001). The development of a safer, more accurate diagnostic tool would revolutionize the treatment of CNS tumors.

Similarly, the current paradigm of clinical and radiographic tumor monitoring during and following treatment is ineffective in guiding clinical decision-making due to radiographic changes with poor sensitivity and specificity for true tumor growth. So-called pseudoprogression–radiographic tumor growth and/or clinical deteriorationis a result of treatment-effect: Inflammatory changes which can be controlled with steroids (Thust et al., 2018). A reliable, non-invasive method for differentiation between progression and pseudoprogression would obviate the need for repeat biopsy and facilitate early treatment.

Histopathologic criteria once dominated brain tumor diagnosis, however, the newer WHO guidelines are progressively shifting toward molecular and genomic parameters (Louis et al., 2016; Kurokawa et al., 2022). A comprehensive method for DNA-methylation-based classification has been established and shown to correct diagnosis in up to 12% of prospective cases (Capper et al., 2018). While imaging fails to capture the genetic tumor profile, liquid biopsy of blood, urine, or cerebrospinal fluid (CSF) for isolation of genetic biomarkers including ctDNA, RNA, and tumor-associated proteins is an emerging method for diagnosis and monitoring of CNS malignancies (Wadden et al., 2022).

Given its ability to transcend the safety concerns and diagnostic limitations of stereotactic biopsy, the development and application of liquid biopsy in clinical CNS tumor management is critical. In this review, we will discuss the latest technologies in biofluid isolation and interpretation, as well as previously identified biomarkers for detection and tracking of various pediatric CNS tumors. Finally, we will discuss the applicability of these techniques and future integration into the current clinical system.

## Amplification and analytic methodologies

Due to a lower total biofluid volume in pediatric patients, the ability to isolate and identify genetic and molecular components from small samples is crucial. Here we will discuss general principles of liquid biopsy in pediatric brain tumors and showcase the feasibility of tumor detection using biofluids (as seen in Table 1). Blood (plasma or serum), urine, and CSF have been identified as potential liquid biopsy candidates in pediatric patients with gliomas (primarily diffuse intrinsic pontine glioma) and embryonal neoplasms (medulloblastoma).

**TABLE 1:** A summary of current liquid biopsy detection methods using varied biofluids, tumor types, and targets.

References	Marker	Detection method	Biofluid type	Tumor type	Targets	Sensitivity
Cantor et al. (2022)	ctDNA	ddPCR	CSF/Plasma	DIPG	H3.3K27M	Plasma: 85.4%
						CSF: 96.5%
Miller et al. (2022)	ctDNA	MSK-IMPACT assay (Illumina)	CSF	pHGG, LGG, MB, PB, DLGNT, RB, E	Genomic coverage ranged from 2x to 1,368x of various somatic alterations	CSF: 46.9%
Pages et al. (2021)	ctDNA	Illumina (ultra-low-pass WGS)	Plasma, Urine, CSF	MB, LGG, LGNT, DIPG, pHGG	10,000x Coverage of 300 Cancer-Related Genes	CSF: 30%
						Plasma: 2.7%
						Urine: 0%
Liu et al. (2021)	ctDNA	Illumina (Low-Coverage WGS)	CSF	MB	CNV: 10q	Metastatic CSF: 85%
					PIM1 SMAD2	Localized CSF: 54%
Li J et al. (2020)	ctDNA	Methylation Sequencing (Illumina HumanMethylation450 BeadChip)	CSF	MB	5 hmC-enriched regions	—
Bruzek et al. (2020)	ctDNA	Nanopore	CSF	pHGG	multiple targets	CSF: 85%
Pan et al. (2019)	ctDNA	Illumina (Deep-Sequencing)	CSF/Plasma	DIPG, MG	Various Targets	Plasma: 38%
						CSF: 100%
Sobol-Milejska et al. (2017)	Protein	ELISA	Whole Blood	MB, AE, pHGG, PB	VEGF/bFGF Levels	—

DIPG, diffuse intrinsic pontine glioma; MG, medullary glioma; pHGG, pediatric high grade glioma; LGG, low grade glioma; MB, medulloblastoma; AE, anaplastic ependymomas; PB, pineoblastomas; DLGNT, diffuse leptomeningeal glioneuronal tumor; LGNT, leptomeningeal glioneuronal tumor; RB, retinoblastoma; E, ependymoma; AST, astrocytoma; AG, astrogliosis; OD, oligodendroglioma; MG, meningioma; DG, diffuse glioma; ctDNA, circulating-tumor DNA; WGS, whole genome sequencing; miRNA, microRNA; CSF, cerebrospinal fluid; ddPCR, droplet digital polymerase chain reaction; RT-qPCR, quantitative reverse transcription polymerase chain reaction.

### Digital droplet PCR (ddPCR)

PCR (polymerase chain reaction) amplifies DNA using a combination of primers, dNTP, and DNA polymerase. Specifically, quantitative PCR captures real-time amplification of targets using fluorescently assigned probes. ddPCR furthermore allows for unbiased amplification of a known target by isolating reactions in each of thousands of droplets (Martínez-Ricarte et al., 2018; Panditharatna et al., 2018). ddPCR is a sensitive and accurate method that can detect rare ctDNA mutations with a limit of detection of approximately .001% in CNS tumors (Hindson et al., 2011; Izquierdo et al., 2021; Li et al., 2021). To demonstrate the utility of this technique, one group used blood ddPCR to amplify ctDNA and found that it was possible to detect the H3.3K27M mutation in 85% of diffuse intrinsic pontine glioma (DIPG) patients at diagnosis and 100% of patients post-radiation or during therapy (Mueller et al., 2019).

### Next-generation sequencing (NGS)

ddPCR can only detect known targets *via* the addition of targeted primers and probes. NGS circumvents this issue with its capacity to seek innumerable unknown genetic alterations (Buermans and den Dunnen, 2014).

NGS (e.g, Illumina) sequences by synthesis, in which a 500-base-pair adaptor is ligated and fluorescent dNTPs are added to the strand to determine the sequence of the target strand (Voelkerding et al., 2009; Buermans and den Dunnen, 2014; Fox et al., 2014). Illumina can be used for whole-genome, whole-exome, or targeted generation sequencing. Results are obtained between 29 h and 4 days (Kanzi et al., 2020). A recent study utilized the NGS Memorial Sloan Kettering-Integrated Molecular Profiling of Actionable Cancer Targets (MSK-IMPACT) assay. Using CSF from pediatric patients with varied histologies and a sequencing depth of 2x to 1,368x, researchers screened patients with existing somatic alterations (Miller et al., 2022).

Nanopore sequencing utilizes miniature pores to sequence strands of DNA (Feng et al., 2015). As a strand of DNA passes through the pore, an electrical current is generated that base-calls nucleotides to sequence the strand. The MinION, a handheld nanopore device, is fast and more affordable than NGS with the ability to reuse parts. The nanopore device can provide same-day results and allows users to make genetic calls within the first few minutes of sequencing (Euskirchen et al., 2017).

### Liquid chromatography mass spectrometry (LC-MS)

Proteomic characterization of CNS tumors is possible *via* liquid chromatography, which separates compounds in a sample based on interactions between mobile and stationary phases. Mass spectrometry is then utilized to convert molecules to the ionized state allowing compound identification by measuring the speed of movement through a vacuum chamber (Pitt, 2009). With only 10 μL of plasma, 862 proteins were identified within 12 h using LC-MS (Xue et al., 2018). Using CSF LC-MS, six proteins were successfully identified that could discriminate the metastatic status of CNS tumors in children compared to controls (Spreafico et al., 2017).

LC-MS can also be used to analyze the metabolites in CSF created by the intake of anti-cancer drugs to assess penetrance beyond the blood-brain barrier (BBB), important for identifying new therapies for clinical trials. A study of seven oral anti-cancer drugs in pediatric CNS tumor patients successfully demonstrated the ability of LC-MS to identify medications with the highest BBB penetrance (Guntner et al., 2020).

## Biomarkers

The current state of the utility of DNA, RNA, and extracellular vesicles in pediatric liquid biopsy samples is reviewed in this section. Four recent seminal papers in the use of liquid biopsy in pediatric brain tumors are discussed in detail (Liu et al., 2021; Cantor et al., 2022; Miller et al., 2022; Pagès et al., 2022).

### DNA

Cell-free DNA (cfDNA) are 50–200 base-pair DNA fragments that are released from cells *via* apoptosis, necrosis, and budding DNA release (Stroun et al., 2001). Circulating tumor DNA (ctDNA) are mutation-carrying fragments released by tumor cells. Both total cfDNA quantification and the mutations identified in ctDNA can be used as biomarkers for tumor diagnosis and progression. Due to its short half-life of 16 min to 2.5 h cfDNA could be a real-time marker of treatment effect and measurable residual disease (MRD) (Mayo et al., 2022).

Due to the high cell turnover rate in malignancies, elevated levels of overall cfDNA are often observed in CSF (Bagley et al., 2020; Nakamura et al., 2020). A study of brainstem gliomas including DIPG demonstrated the ability to reliably detect primary tumor alterations in CSF ctDNA (Pan et al., 2019). Our group demonstrated correlation between the concentration of characteristic mutant K27M copies in CSF ctDNA and contrast-enhancing tumor area on MRI, as well as with cell proliferation in an *in vitro* model (Stallard et al., 2018). We further applied this technique in a larger cohort as part of a prospective clinical trial, demonstrating that change in H3.3K27M variant allele fraction (VAF) in both CSF and plasma was associated with increased progression-free survival. VAF increase of >25% was found to precede tumor progression in many cases, and cases of radiographic progression without an increase in VAF were later found to reflect pseudo-progression. These data show that CSF or plasma ctDNA H3.3K27M VAF could be a reliable biomarker for prediction of progression and for differentiation from pseudoprogression (Cantor et al., 2022).

When CSF was collected serially in children with medulloblastoma (MB), low-coverage WGS detected MRD in 54% and 85% of patients with localized and metastatic disease, respectively (Liu et al., 2021). Among 64 CSF samples from a diverse range of pediatric and young adult patients, cfDNA positivity correlated strongly with disseminated disease (Miller et al., 2022). While these data are promising, a large-scale attempt to standardize liquid biopsy for DNA biomarker isolation across multiple tumor histologies using ultra-low pass NGS was fairly unsuccessful with copy number alterations only detected in 20% of CSF samples and fewer in other biofluids (Pagès et al., 2022).

### RNA

microRNAs (miRNAs) are non-coding molecules 18–24 nucleotides in length (Eibl and Schneemann, 2021). They are integral to the stability and translation of messenger RNA (mRNA) and can impact tumor angiogenesis, growth, and invasiveness (Floyd and Purow, 2014). miRNAs have been isolated from CSF, blood, and urine and are stable in biofluids. However, the standardization of control and tumor-specific miRNA panels is in its infancy.

Considering the difficulty in obtaining CSF specimens from children, liquid biopsy of serum and urine miRNA in pediatric brain tumor patients would be preferred, however, there is a relative paucity of information in this subject. Serum miRNA in pediatric astrocytomas has been found to demonstrate characteristic miRNA signatures; miRNA levels were used to successfully predict tumor size and therapeutic response (López-Aguilar et al., 2017; Bookland et al., 2018). Furthermore, reverse transcription of cell-free tumor RNA into complementary DNA has successfully detected *EGFR(v)III* amplification in pediatric high-grade gliomas (Figueroa et al., 2017; Manda et al., 2018).

In MB (most common cerebellar tumor in pediatric patients) miRNA expression changes have been identified that directly regulate tumorigenic MB pathways including SHH and WNT (Braoudaki and Lambrou, 2015; Wang et al., 2022). These biomarkers could provide MB genetic classification without surgical biopsy.

### Extracellular vesicles

Extracellular vesicles (EV) are small, membrane-bound structures secreted from both normal and cancerous cells. EVs have been found to hold tumor-specific DNA, various RNAs, and proteins (Doyle and Wang, 2019). Because of their small size and tumor-specific cargo, EVs have been suggested as a biomarker for liquid biopsy in adult gliomas (Nikoobakht et al., 2022).

The diagnostic potential of EVs in pediatric brain tumors is not well studied. Proteomic signatures isolated from pediatric medulloblastoma cell lines and patient sera revealed both a brain tumor-specific profile as well as a MB-specific profile (Epple et al., 2012). Furthermore, spike-in media-derived exosomes were found to be cell attractants, increasing cell migration *in vitro*. Isolation of EVs from the cell media supernatant of 3 DIPG and 4 MB cell lines identified EV-derived miRNAs unique to DIPG and MB respectively (Magaña et al., 2022). Additionally, the study identified EV-specific miRNAs when compared to miRNAs isolated from parent tumor cells, suggesting targeted loading of miRNAs into EVs.

Further research may unlock important mechanistic discoveries and therapeutic targets. However, the lack of standard protocols for EV isolation and lack of liquid patient samples are barriers to further discovery.

## Clinical applications

**FIGURE 1:**
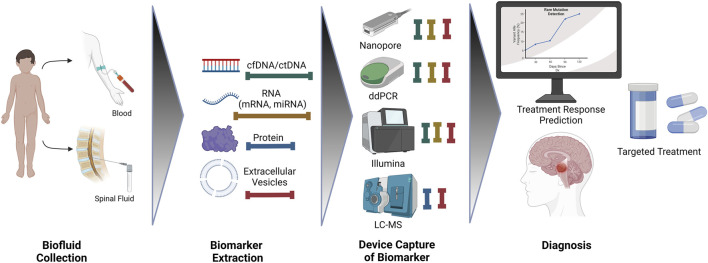
An overview of biofluid collection, extraction, detection, and diagnosis for improved treatment outcomes.

Above we reviewed methods used to study transcriptomic and proteomic signatures of pediatric CNS tumors in biofluids. Next, we will discuss the utilization of liquid biopsy in practice, including applications in diagnosis and surveillance treatment as seen in Figure 1.

### Diagnosis/molecular characterization

Genomic profiling of pediatric brain tumors has identified targetable mutations in more than half (Ramkissoon et al., 2017; Cole et al., 2018). In high-risk pediatric glial tumors, exome and transcriptome sequencing prompted a change in therapy in more than two-thirds (Koschmann et al., 2018). This has further been shown to be reproducible using liquid biopsy, with the ability to identify targetable mutations, monitor therapeutic response, and follow tumor evolution (Miller et al., 2022). Molecular profiling is now recommended along with IHC to guide care in pediatric glioma patients (Miklja et al., 2019). Spatial subclones with unique genotypes and phenotypes have been identified in pediatric GBM and DIPG (Vinci et al., 2018; Harpaz et al., 2022) limiting the accuracy of a single stereotaxic biopsy. Liquid biopsy includes elements secreted from all parts of the tumor, with better representation that can provide a more accurate diagnosis.

Liquid biopsy could also facilitate early minimally invasive tumor identification, avoiding treatment delays where appropriate. Of incidentally discovered pediatric brain tumors, most are monitored with imaging and 10% eventually require treatment (Soleman et al., 2020). In an adult diffuse glioma pilot study, 85% of tumors were successfully diagnosed *via* CSF ctDNA sequencing with a 7–10 days turnaround (Martínez-Ricarte et al., 2018). However, when specimens were prospectively collected in 564 children, copy number alterations were only detected in 20% of CSF, 1.3% of plasma, and 0% of urine samples (Pagès et al., 2022). In the setting of low ctDNA levels in children and low levels of clonal aberrations, extensive further research in the pediatric population is necessary to develop ctDNA as a biomarker and lumbar puncture as an early diagnostic option.

Several pediatric CNS tumors are difficult to diagnose even using tumor morphology, imaging, immunohistochemistry, sequencing, and FISH. Of these diagnostic enigmas, 71% were successfully diagnosed using DNA methylation profiling, a parameter easily accessible using liquid biopsy nucleic acids (Pages et al., 2021). Liquid biopsy miRNA markers for medulloblastoma have been identified for differentiation from controls with high specificity (Wang et al., 2022). In primary CNS lymphoma, the DNA yield, variant allele fraction, and ability to detect MYD88 (a characteristic mutation) were actually much better in CSF cfDNA than in stereotactic biopsy cellular DNA, the current gold standard for diagnosis (Yamagishi et al., 2021).

Intraoperatively, the MinION nanopore device identifies genetic data within the first few minutes of sequencing using fresh frozen tumor tissue (Euskirchen et al., 2017), which could dictate extent-of-resection decision-making. Though Nanopore has previously been limited by a higher error rate as compared to NGS, our group demonstrated 85% sensitivity and 100% specificity for ctDNA in CSF samples from pediatric high-grade glioma patients (Slatko et al., 2018; Bruzek et al., 2020).

### Treatment response prediction/surveillance

During and following treatment of brain tumors, patients are monitored with serial MRI imaging which often fails to differentiate true progression from pseudo-progression. Serum biomarkers have been used to monitor progression in other cancers (i.e. PSA in prostate cancer, CEA in colorectal cancer, CA125 in ovarian cancer, AFP in hepatocellular carcinoma, and beta-hCG in gestational trophoblastic disease). A correlate in pediatric brain cancers could play an important role in post-treatment monitoring and early treatment of recurrence. Serial liquid biopsy in addition to serial MRI may reveal new genetic targets for precision chemotherapy (Kim et al., 2015; Salloum et al., 2017).

Our group demonstrated that change in CSF cfDNA H3K27M variant allele fraction predicted treatment response and tumor progression following ONC201 treatment in diffuse midline glioma (Cantor et al., 2022). The same biomarker was also found to be correlated with treatment response to radiotherapy by another group (Panditharatna et al., 2018). In medulloblastoma, CSF cfDNA was used as a means to detect MRD, demonstrating that patients with persistent copy number alteration detection were at higher risk of progression. Furthermore, marker detection preceded radiographic progression in half of relapsing patients (Liu et al., 2021). DNA methylation status in CSF ctDNA has also been identified as an epigenetic prognostic marker to predict outcomes in MB (Li J et al., 2020).

## Conclusion/future directions

Brain biopsy, the gold standard of pediatric brain tumor diagnosis, is a risky procedure with unreliable diagnostic value. Liquid biopsy could be a minimally invasive, definitive method to diagnose, monitor, and treat these lethal cancers. CSF procurement *via* lumbar puncture in children is complicated. Furthermore, it has been shown that CSF sampling more proximal to an intracranial tumor may have increased sensitivity for CSF ctDNA detection (Stallard et al., 2018). Studies have also found that placement of a fourth ventricular catheter for even greater proximity to a posterior fossa tumor is safe (Sandberg and Kerr, 2016).

LP is the safest, least invasive method to obtain CSF during initial brain lesion workup. However, in patients who undergo surgical resection, an alternative CSF procurement method could involve placement of an Ommaya reservoir and peritumoral or intraventricular catheter. Lateral and third ventricular catheters are routinely placed by neurosurgeons for CSF diversion or intrathecal chemotherapy. Still, reservoir placement for tumor monitoring and CAR-T therapy in pediatric patients has only recently become standard of care (Vitanza et al., 2021).

Given that most pediatric tumors present in the posterior fossa, hydrocephalus is a common issue. More than 60% of children with DIPG require surgical intervention for relief, many of whom require shunt placement. In a review of several studies of shunt catheters in DIPG, the overall infection rate was <1% (Li D et al., 2020). In children requiring ventricular Ommaya reservoir for CSF chemotherapy delivery, only 1% developed an infection requiring explantation (Peyrl et al., 2014). Other rare complications include hemorrhage during placement, clogging, and superficial local CSF effusion. Given safety of placement, the potential benefits for longitudinal disease monitoring and treatment guidance far outweigh the benefits.

Blood sampling is innocuous when compared with CSF extraction. The sensitivities of sequencing technologies used to study biofluids, specifically low-coverage WGS and targeted Nanopore NGS, are increasingly improving. As the ability to detect nucleic acid and proteomic biomarkers from liquid biopsies improve, the emphasis will transition from CSF to serum studies. We have already begun to demonstrate that plasma can be used to direct clinical decision-making without awaiting identifiable radiographic progression (Cantor et al., 2022).

Further research is required in the characterization and standardization of biomarkers found in CSF and other biofluids, which could represent the next paradigm shift in clinical management of pediatric brain tumor patients.
